# Exploring the genetics of trotting racing ability in horses using a unique Nordic horse model

**DOI:** 10.1186/s12864-019-5484-9

**Published:** 2019-02-04

**Authors:** Brandon D. Velie, Mette Lillie, Kim Jäderkvist Fegraeus, Maria K. Rosengren, Marina Solé, Maja Wiklund, Carl-Fredrik Ihler, Eric Strand, Gabriella Lindgren

**Affiliations:** 1 Department of Animal Breeding and Genetics, Swedish University of Agricultural Sciences, Uppsala, Sweden; 2 School of Life and Environmental Sciences, University of Sydney, Sydney, Australia; 3 Department of Biological and Environmental Sciences, University of Gothenburg, Gothenburg, Sweden; 4 Department of Ecology and Genetics, Uppsala University, Uppsala, Sweden; 5 Department of Clinical Sciences, Swedish University of Agricultural Sciences, Uppsala, Sweden; 6 Department of Companion Animal Clinical Sciences, Norwegian University of Life Sciences, Oslo, Norway; 7 Livestock Genetics, Department of Biosystems, KU Leuven, Leuven, Belgium

**Keywords:** Athleticism, Conformation, Genomic, Performance, Racehorse

## Abstract

**Background:**

Horses have been strongly selected for speed, strength, and endurance-exercise traits since the onset of domestication. As a result, highly specialized horse breeds have developed with many modern horse breeds often representing closed populations with high phenotypic and genetic uniformity. However, a great deal of variation still exists between breeds, making the horse particularly well suited for genetic studies of athleticism. To identify genomic regions associated with athleticism as it pertains to trotting racing ability in the horse, the current study applies a pooled sequence analysis approach using a unique Nordic horse model.

**Results:**

Pooled sequence data from three Nordic horse populations were used for F_ST_ analysis. After strict filtering, F_ST_ analysis yielded 580 differentiated regions for trotting racing ability. Candidate regions on equine chromosomes 7 and 11 contained the largest number of SNPs (*n* = 214 and 147, respectively). GO analyses identified multiple genes related to intelligence, energy metabolism, and skeletal development as potential candidate genes. However, only one candidate region for trotting racing ability overlapped a known racing ability QTL.

**Conclusions:**

Not unexpected for genomic investigations of complex traits, the current study identified hundreds of candidate regions contributing to trotting racing ability in the horse. Likely resulting from the cumulative effects of many variants across the genome, racing ability continues to demonstrate its polygenic nature with candidate regions implicating genes influencing both musculature and neurological development.

**Electronic supplementary material:**

The online version of this article (10.1186/s12864-019-5484-9) contains supplementary material, which is available to authorized users.

## Background

As genomics improves and enables the design of more targeted studies relating genotypes to phenotypes, the opportunity for non-model organisms continues to expand - facilitating greater opportunities to gain novel insight into the mechanisms regulating biological homeostasis and health [1, 2]. Genomic studies of natural model species, domestic species in particular, give a complimentary view of genotype-phenotype relationships compared with the knowledge gained from the study of humans and experimental organisms [2]. Since the onset of domestication, horses have been strongly selected for, among other things, speed, strength, and endurance-exercise traits [3]. This diverse and, at times, divergent selection has ultimately led to the development of highly specialized horse breeds. Within the last 400 years, breed specialization has focused primarily on preserving and improving traits related to aesthetics and performance [3]. As a result, most horse breeds today are closed populations with high phenotypic and genetic uniformity within breed. However, a great deal of variation continues to exist among breeds [3]. This variation, combined with breed specialization, has made the horse particularly well suited for genetic studies of locomotion patterns and provides a unique opportunity for genetic studies of athleticism [1–5]. Generally speaking, athleticism describes the physical qualities that are characteristic of athletes and typically refers to traits such as strength, fitness, and agility. Many modern day horse breeds exemplify some if not all of these traits, with shared selective pressures within breeds (e.g. health, fertility traits, conformation) and divergent selection between breeds (e.g. speed vs strength) yielding a wide range of athletic phenotypes [3, 6].

**Fig. 1:**
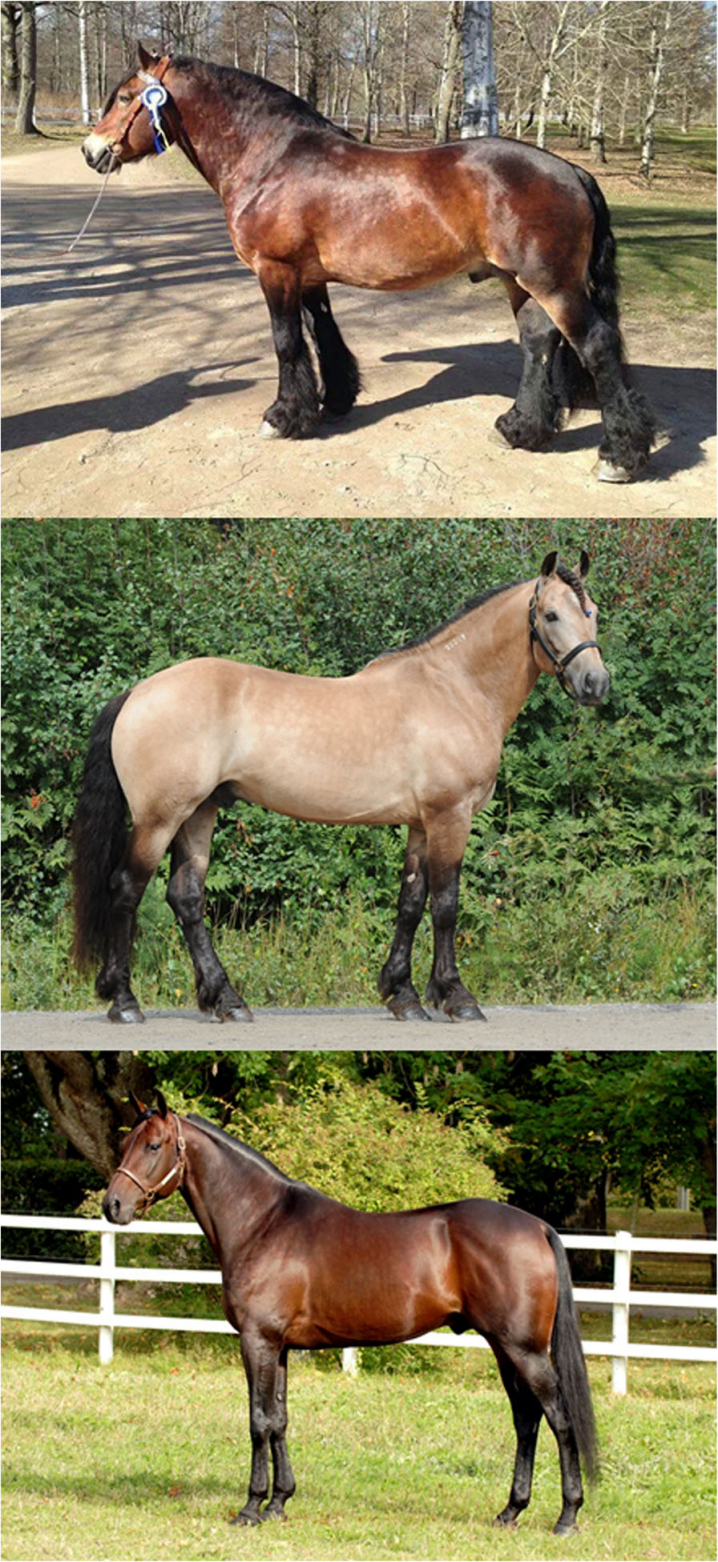
Breed classifications. Top panel = North Swedish Draught Horse; middle panel = Norwegian-Swedish Coldblooded trotter; bottom panel = Standardbred trotter. *Images used in the figure are privately owned and thus were not taken from previously published sources requiring written permission for use

Here we apply a genomic approach to investigate athletic phenotypes associated with trotting racing ability (TRA) using a unique Nordic horse model consisting of the Norwegian-Swedish Coldblooded trotter (NSCT), the North Swedish Draught horse (NSD), and the Standardbred trotter (SB) (Fig. 1). Although both the NSCT and the NSD are horse breeds derived from the original North-Swedish horse, a small, heavy horse traditionally used in agriculture and forestry work, selection for traits beneficial to agricultural work only continues in the NSD [7–11]. Since the 1960s, the NSCT has been intensively selected for harness racing performance and is now considered a true racing breed [10–13]. As a result, a remarkable improvement in the racing performance of NSCTs has occurred during the last half-century. However, it is also well established that some degree of cross-breeding occurred between NSCT and SBs, a significantly faster breed of horse from a different gene pool, before obligatory paternity testing was introduced in Sweden in 1969 [10–13]. Consequently, the improvement in NSCT racing performance may be partially explained by a marked increase of favorable genetic variants originating from SBs. It is this specific attempt at gaining a competitive racing advantage that makes the NSCT ideal for genomic studies investigating TRA phenotypes.

Despite a dispersed history of crossbreeding with SBs, the relationship between the NSCT and NSD remains closer than either of the breeds with the SB [10]. While both the NSCT and the SB are selected for racing performance, the Norwegian and Swedish breed organizations have remained highly committed to preserving the historical work-horse appearance of the NSCT breed [7, 8]. As a result, both NSCTs and NSDs can be classified as heavy horse breeds, with NSCTs sometimes referred to as “draft trotters” (Fig. 1) [14]. Any lingering genetic similarities between the NSCT and the SB are therefore highly likely to be associated with favorable traits for TRA. Our aim was to identify these similarities using pooled whole genome sequence data from a carefully selected sample of NSCTs, NSDs, and SBs.

## Results

A summary of the population statistics for the three populations is provided in Tables 1 and 2.

**Table 1:** Pooled population samples and genome information

Breed	Number of individuals^a^	Coverage	Nucleotide diversity (π) (%)^b^
Norwegian-Swedish Coldblooded trotter	18	43.98	0.171
North Swedish Draught	25	35.96	0.175
Standardbred trotter	22	56.26	0.159

^a^Male/Female ratio: Norwegian-Swedish Coldblooded trotter = 2/1; North Swedish Draught = 25/0; Standardbred trotter = 7/4

^b^Nucleotide diversity calculated over 5000 bp windows, averaged across the genome (%)

**Table 2:** Differentiation between breeds

	NSD	NSCT	SB
NSD		0.07159	0.11077
NSCT	0.05755		0.10819
SB	0.08823	0.08555	

Mean F_ST_ above the diagonal, median F_ST_ below the diagonal

*NSD* North Swedish Draught Horse, *NSCT* Norwegian-Swedish Coldblooded Trotter, *SB* Standardbred trotter

### F_*ST*_ analyses

**Fig. 2:**
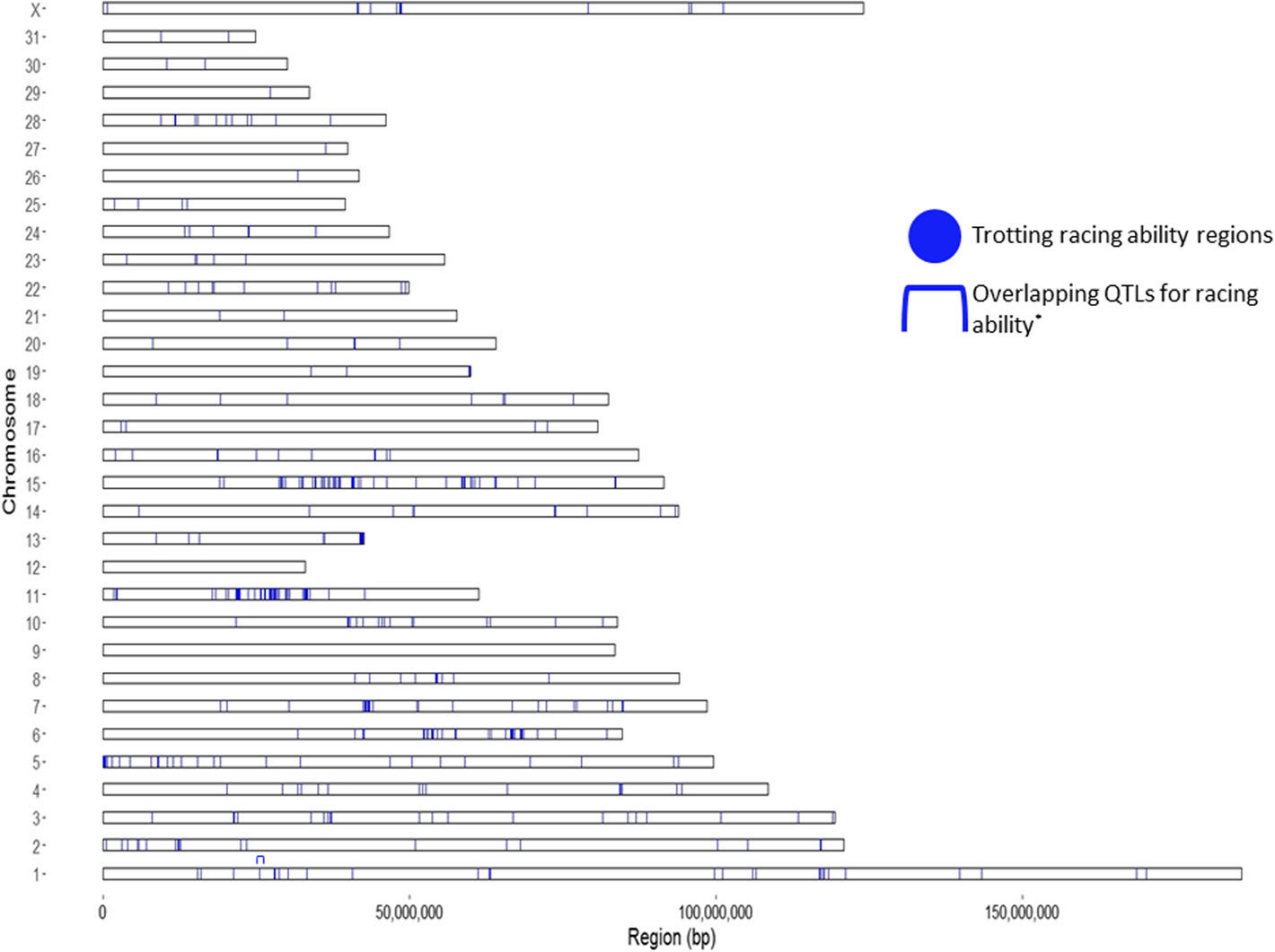
Locations of highly differentiated genomic regions across the horse chromosomes. *Ricard et al. 2017

After clustering of windows located less than 0.1 Mb apart and filtering out regions containing less than 2 SNPs or windows measuring only 1000 bp, 580 differentiated regions were retained (Fig. 2; Additional file 1). Candidate regions ranged in length from 1.5 kb to 773.5 kb (mean/median length: 80/56 kb; total cumulative length: 46.389 Mb).

**Fig. 3:**
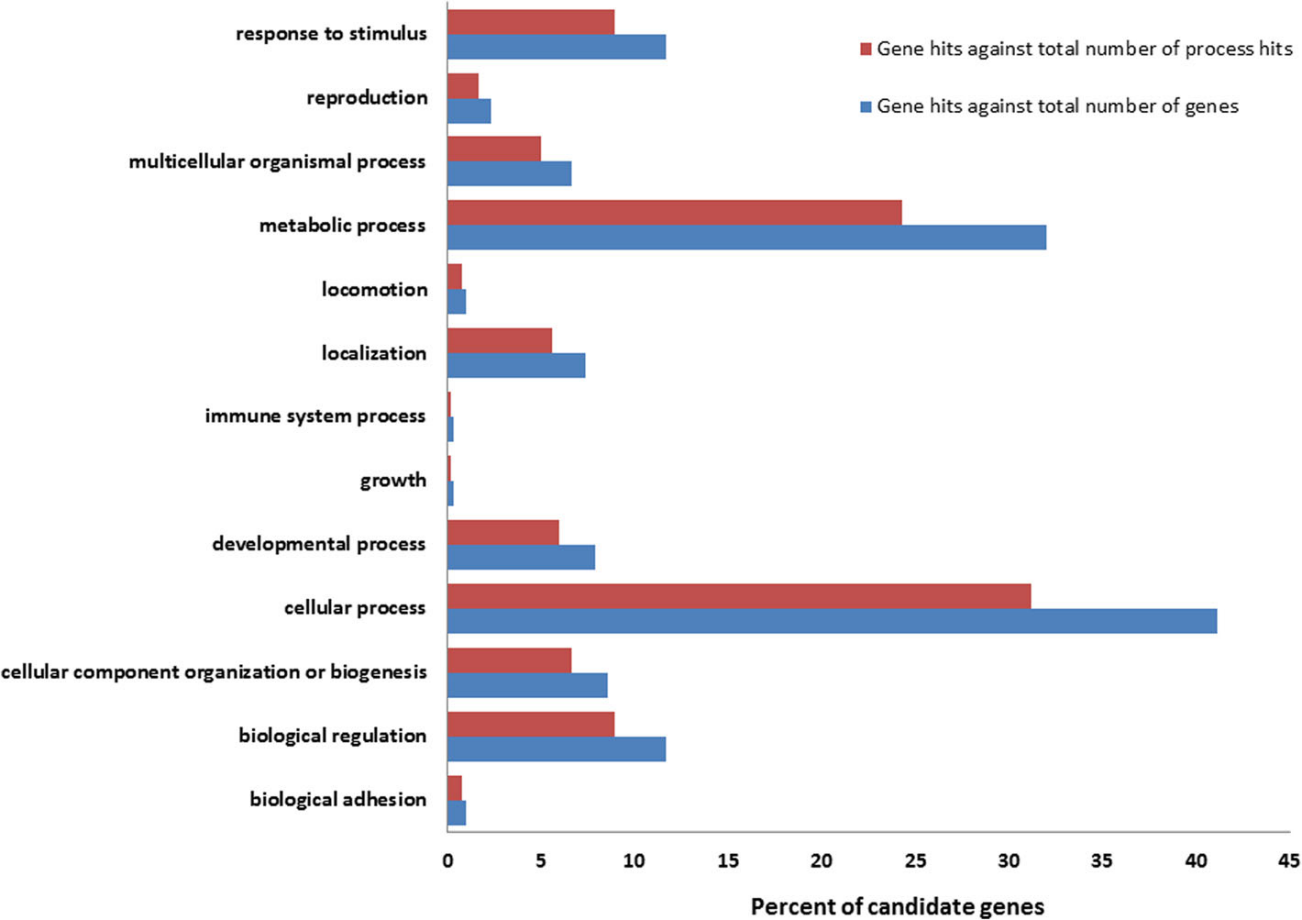
Molecular function summary information from the functional classification analysis of candidate genes in PANTHER. PANTHER molecular function classification: the function of the protein by itself or with directly interacting proteins at a biochemical level

**Fig. 4:**
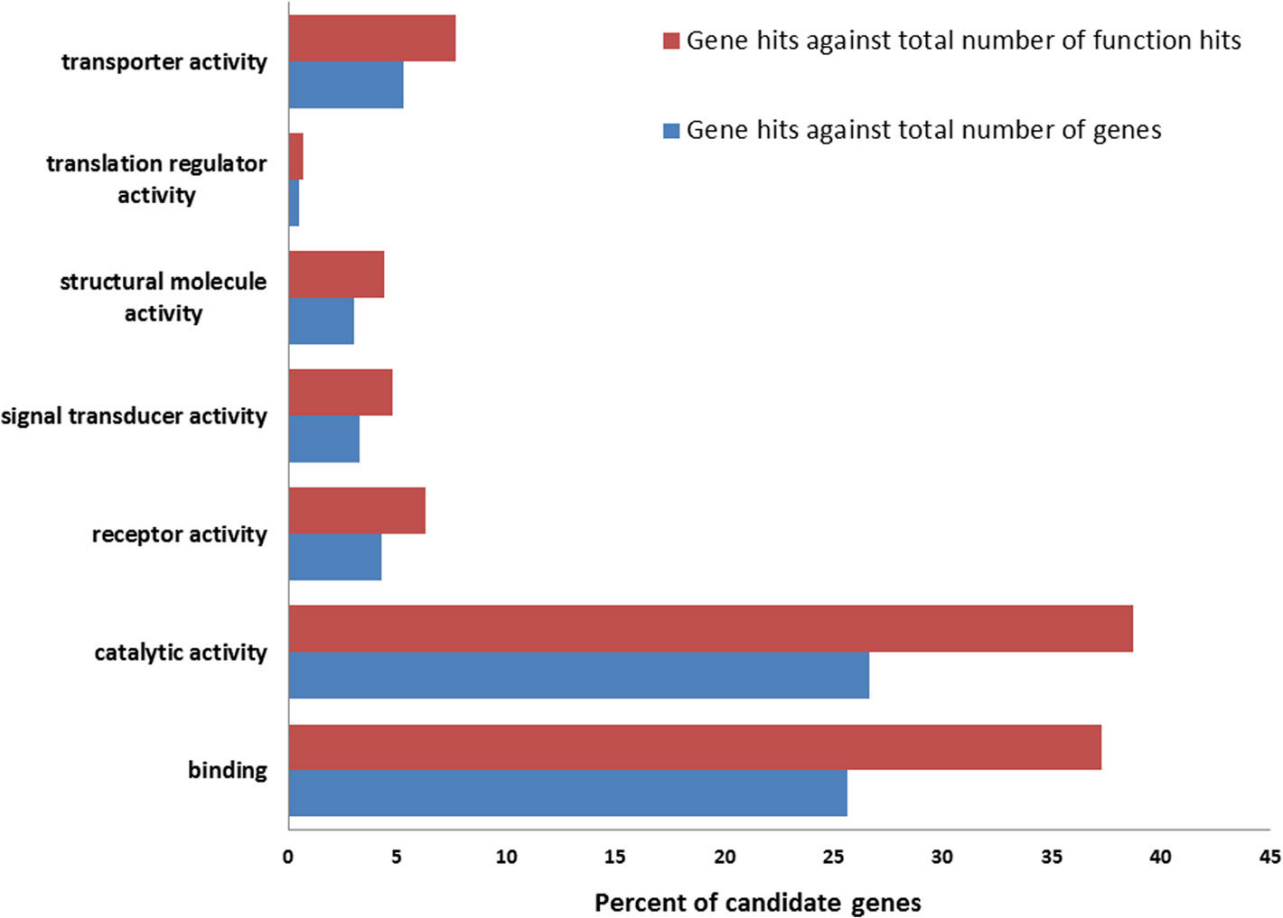
Biological process summary information from the functional classification analysis of candidate genes in PANTHER. PANTHER biological process classification: the function of the protein in the context of a larger network of proteins that interact to accomplish a process at the level of the cell or organism

Candidate regions on *Equus caballus* chromosome (ECA) 7 and 11 contained the largest number of SNPs at 214 (length: 151 kb) and 147 (length: 683.5 kb), respectively. Multiple regions on ECA 11, 13, and 15 were greater than 500 kb in length (Fig. 2; Additional file 1). From GO analysis, candidate regions identified contained 271 candidate genes associated with known molecular functions, 519 with known biological processes, and 124 with known pathways (Figs. 3 and 4; Additional files 2 and 3). One candidate region for TRA overlapped with a previously characterized QTL for racing ability [15]. This region was located on ECA 1 and overlapped a QTL for racing speed (Fig. 2; Table 3).

**Table 3:** QTLs overlapped by trotting racing ability candidate regions

QTL ID	ECA	Start Position	End Position	Trait Name	Study
138,687	1	25,715,314	25,715,354	racing speed	Ricard et al. 2017

*ECA Equus caballus chromosome*

## Discussion

For all intents and purposes, racehorses are professional athletes. Like professional human athletes, racehorses must not only endure the day to day physical demands of their sport, but they must also have a genetic capacity for athleticism relative to their sport in order to ultimately achieve success (i.e. win). However, unlike in humans, racehorses have been carefully selected and bred for centuries, resulting in alleles with subtle effects on athleticism being enriched over time. As a result, racehorses in particular provide a unique opportunity to identify genes and subsequently the molecular mechanisms underpinning athletic ability. Rarely found outside of the Nordic countries, the NSCT is perhaps one of the most unique types of racehorse in the modern era – originating not from historic racing breeds such as the Thoroughbred or Standardbred, but instead tracing its lineage back to the original North Swedish horse [10, 11]. Using whole-genome re-sequencing of pooled DNA from a carefully selected group of NSCTs, NSDs, and SBs, we capitalized on this unusual ancestry of the NSCT and identified 580 candidate regions for TRA in the horse.

Only one previously characterized QTL for racing ability was overlapped by the TRA candidate regions in the current study [15]. A SNP in the first intron of the sortilin related VPS10 domain containing receptor 3 (*SORCS3*) gene, previously associated with racing speed in endurance horses, was overlapped by a 107.5 kb candidate region on ECA1 [15]. The gene encodes for a type-I transmembrane receptor protein that is a member of family of receptors with known pleiotropic functions [16]. Genetic variation in *SORCS3* has been associated with Alzheimer’s disease in humans with more recent studies suggesting that additive epistatic effects of genetic variants within the gene may be important [17–19]. Furthermore, variation in *SORCS3* has been associated with attention deficit hyperactivity disorder and *SORCS3* knockout mice display defects in spatial learning and memory, as well as increased fear extinction [20, 21]. Although the function of this gene in horses remains unknown, as the transcript is generally expressed at high levels in the brain, it could perhaps alter an individual’s perception of athletic competition (e.g. altered feedback loop in response to exercise, reduced fear extinction) [20].

Interestingly, while a mere 1.9% (46.39 Mb) of the genome was covered by TRA candidate regions, the regions on ECA11 not only accounted for 11.2% of all TRA candidate regions identified, but ECA11 candidate regions also contained some of the highest number of SNPs (*n* = 100+) and the largest sweeps (> 50 kb in length). Furthermore, ECA11 had the highest concentration of candidate TRA regions when compared to the other equine chromosomes. However, despite the density of this TRA signal, no QTLs for racing ability have been mapped to ECA11 [22].

Consequently, regions on ECA11 have previously been associated with size (i.e. height and mass), which, given the prudent design of the current study, is particularly interesting [3, 23, 24]. Although similar in height, NSCTs, NSDs, and SBs differ in their physique. SBs tend to be leaner and more refined in their appearance compared with NSCTs, while NSCTs tend to be leaner and more refined than NSDs (Fig. 1). In order for a region to have been considered as a candidate region for TRA in the current study, the region had to be highly similar between NSCTs and SBs yet decidedly different from NSDs. It is possible that strict adherence to include only top performing NSCTs in the sequenced pool may have skewed the NSCT pool towards lighter framed horses; thereby demonstrating what conceivably is a competitive racing advantage for lighter horses. Perhaps even more interesting to note is that no previously reported QTLs for growth or conformation traits overlapped any of the candidate regions for TRA on ECA11; however, a recent study in American Quarter horses also suggested ECA11 as potentially important for racing ability [25].

ECA11 also contained the candidate region with the second largest number of SNPs (*n* = 147) in the study. The region, located ECA11:32,874,000-33,557,500, encompasses 9 genes, (RAD51C, PPM1E, ENSECAG00000003590, ENSECAG00000015244, GDPD1,YPEL2, SKA2, PRR11) one of which is tripartite motif containing 37 (*TRIM37*). Mutations in *TRIM37* are associated with Mulibrey Nanism in humans, an extremely rare autosomal recessive disorder characterized by profound growth delays and abnormalities of the muscles, liver, brain, and eyes [26, 27]. Instinctively this would further support the candidate region being associated with body size; however, even if this is the case, it does not necessarily mean the region is solely associated with body size and shape. It is highly plausible that haplotypes associated with body size differ by multiple substitutions with pleiotropic functional effects. Mutations that impact underlying mechanisms for muscle, ligament, and tendon development would certainly influence TRA – limiting racing ability in some instances, while enhancing racing ability in others [28–30]. Moreover, a large conserved haplotype containing tripartite motif containing 13 (*TRIM13*), a gene located on ECA17, has previously been suggested as having selective importance in the Thoroughbred [3]. *TRIM37*, while located on a different chromosome, is part of the same gene family as *TRIM13*.

## Conclusions

This study identified hundreds of candidate genomic regions contributing to TRA in the horse, a result not unexpected for investigations into the genomics of such a complex trait. The trait is undoubtedly polygenic, resulting from the cumulative effects of many variants across the genome. Candidates for TRA implicated both genes influencing musculature and conformation, as well as genes involved in neurological development, further suggesting that racing ability may not solely be a product of physical characteristics, but also mental characteristics. This study identified a strong racing ability signal on ECA11 that will be particularly interesting for follow-up.

## Methods

### Animals

Genomic DNA samples from 18 NSCTs, 25 NSDs, and 22 SBs were prepared from blood samples and pooled in equimolar ratios prior to library construction (Table 1). Each horse was selected based on strict breed specific criteria. All trotting horses racing in Norway and Sweden have breeding values estimated annually. For NSCTs, estimated breeding values (EBVs) are estimated using an animal model that includes the combined effect of country, sex, and birth year. EBVs are subsequently based on racing performance results (i.e. racing status and earnings) occurring between 3 and 6 years of age [31]. For inclusion in the current study, NSCT horses were required to have an estimated breeding value of at least 115 and sire/progeny ratios were restricted to reflect the larger population as accurately as possible (Additional file 4) [7, 8].

For EBVs in SBs, the animal model includes genetic base group and a combination of sex and birth year with the evaluation based on racing performance results occurring between 2 and 5 years of age [31]. For inclusion in the current study, Standardbreds were also required to have estimated breeding values of at least 115 and both SBs and NSDs were not allowed to have a common ancestor within three generations (i.e. no shared sires, dams, grandsires, granddams) [7, 9]. An EBV requirement for NSDs was not possible as EBVs are not calculated for this breed.

### Pool sequencing, genome alignments, variant calling, and population analyses

Genome sequencing library construction and sequencing was carried out by SciLifeLab (Uppsala, Sweden) using two lanes on the Illumina HiSeq2500 (150 bp paired-end). Sequencing libraries were prepared from 100 ng DNA using the TruSeq Nano DNA sample preparation kit targeting an insert size of 350 bp. Reads were aligned to the *Equus caballus* genome (EquCab2.70) using BWA (v0.7.15) [32]. Duplicates were marked with Picard (v1.118; http://broadinstitute.github.io/picard/) and GATK was used for realignment around indels [33]. Samtools (v1.8 [34, 35]) was used to generate the mpileup files needed for Popoolation (v1.2.2) and PoPoolation2 (v1.201) [36]. Nucleotide diversity (π) was calculated across 5000 bp windows for each population pool using Popoolation [36]. PoPoolation2 was used to calculate F_ST_ over 1000 bp sliding windows with 50% overlap between the selected population samples using the Karlsson et al. method [36, 37]. Minimum count was set at 3, minimum coverage at 10, maximum coverage at 100, and minimum coverage fraction at 1.

### Differentiated regions

Given the close relationship between NSCTs and NSDs, candidate regions for athletic traits were defined as genomic regions where F_ST_ values were relatively high between NSCTs and NSDs, but low between NSCTs and SBs. As such, stringent F_ST_ cutoffs (> 95% percentile, F_ST_ = 0.179 NSCT vs. NSD; < 5% percentile, F_ST_ = 0.013 NSCT vs. SB) were used when defining candidate regions. Windows with F_ST_ values that met these criteria were clustered into candidate sweep regions when they were less than 0.1 Mb from one another (custom R scripts) [38]. Clusters containing only a single 1000 bp window or less than 2 SNPs were excluded. Candidate gene screening was subsequently carried out using the bioinformatics database Ensembl (http://www.ensembl.org/). Candidate regions from the F_ST_ analyses were used to generate a list of annotated genes using the Ensembl Biomart function. The resulting list of candidate genes was then piped into the PANTHER Classification system in order to obtain an overview of the molecular functions and biological processes affected by the candidate genes [39, 40]. Previously reported racing ability QTLs in the horse (downloaded from the horse QTL database; [22]) were also compared to differentiated regions to determine overlaps using bed file comparisons in BEDOPS [41].

## Additional files

Additional file 1: Summary information for trotting racing ability candidate sweep regions. (XLSX 53 kb)

Additional file 2: Functional classification gene list from PANTHER analysis of trotting racing ability candidate sweep regions. (XLSX 42 kb)

Additional file 3: Pathway summary information from the functional classification analysis of trotting racing ability candidate sweep regions. (XLSX 12 kb)

Additional file 4: Pedigree breakdown for the 18 Norwegian-Swedish Coldblooded trotters (NSCT) included in the NSCT pool. (XLSX 11 kb)

## Abbreviations

EBVs: Estimated breeding values
ECA: *Equus caballus* chromosome
GDPD1: Glycerophospodiester phosphodiesterase domain containing 1
NSCT: Norwegian-Swedish Coldblooded trotter
NSD: North Swedish Draught horse
PPM1E: Protein phosphatase Mg2+/Mn2+ dependent 1E
PRR11: Proline rich 11
RAD51C: RAD51 paralog C
SB: Standardbred trotter
SKA2: Spindle and kinetochore associated complex subunit 2
SORCS3: Sortilin related VPS10 domain containing receptor 3
TRA: Trotting racing ability
TRIM13: Tripartite motif containing 13
TRIM37: Tripartite motif containing 37
YPEL2: Yippee like 2
